# An explanatory model of factors related to well baby visits by age three years for Medicaid-enrolled infants: a retrospective cohort study

**DOI:** 10.1186/1471-2431-13-158

**Published:** 2013-10-05

**Authors:** Donald L Chi, Elizabeth T Momany, Michael P Jones, Raymond A Kuthy, Natoshia M Askelson, George L Wehby, Peter C Damiano

**Affiliations:** 1 Department of Oral Health Sciences, University of Washington, Box 357475, Seattle, WA 98195, USA; 2 Public Policy Center, University of Iowa, 210 SQ, Iowa City, IA 52242, USA; 3 Department of Preventive and Community Dentistry, University of Iowa, N329 DSB, Iowa City, IA 52242, USA; 4 Department of Biostatistics, University of Iowa, C22-GH, Iowa City, IA 52242, USA; 5 Department of Health Management and Policy, University of Iowa, E205-GH, Iowa City, IA 52242, USA

## Abstract

**Background:**

Well baby visits (WBVs) are a cornerstone of early childhood health, but few studies have examined the correlates of WBVs for socioeconomically vulnerable infants. The study objective was to identify factors related to the number of WBVs received by Medicaid-enrolled infants in the first three years of life and to present a preliminary explanatory model.

**Methods:**

We analyzed Iowa Medicaid claims files and birth certificate data for infants born in calendar year 2000 (N = 6,085). The outcome measure was the number of well baby visits (WBVs) received by Medicaid-enrolled infants between age 1 and 41 months (range: 0 to 10). An ecological health model and existing literature were used to evaluate 12 observed factors as potential WBV correlates. We ran multiple variable linear regression models with robust standard errors (α = 0.05).

**Results:**

There were a number of infant, maternal, and health system factors associated with the number of WBVs received by Medicaid-enrolled infants. Infants whose mothers had a greater number of prenatal healthcare visits (ß = 0.24 to 0.28; P = .001) or were married (ß = 0.20; P = .002) received more WBVs. Having a chronic health condition (ß = 0.51; P < .0001) and enrollment in a case management program (ß = 0.48; P < .0001) were also positively associated with WBVs. Eligibility for Medicaid through the Supplemental Security Income Program (ß = −0.70; P = .001), increased maternal age (ß = −0.27 to −0.35; P = .004), higher levels of maternal education (ß = −0.18; P = .005), maternal smoking (ß = −0.13; P = .018), and enrollment in a health maintenance organization plan (ß = −1.15; P < .0001) were negatively associated with WBVs. There was a significant interaction between enrollment in a health maintenance organization plan and enrollment in a Medicaid case management program (P = .015). Maternal race, maternal alcohol use during pregnancy, and rurality were not significantly related to the number of WBVs.

**Conclusions:**

Multiple infant, maternal, and health system variables were related to the number of WBVs received by Medicaid-enrolled infants. Additional research is needed to develop strategies to optimize access to WBVs for Medicaid-enrolled infants at risk for poor use of preventive medical care services.

## Background

Well baby visits (WBVs) are a cornerstone of early childhood health as well as disease prevention and management. Defined as “a series of frequent, repetitive, routine examinations” by a medical provider [1], WBVs include monitoring of physical growth, sensory screenings, developmental and behavioral assessments, and immunizations [2]. WBVs are an important conduit through which caregivers receive anticipatory guidance from medical providers and referrals for specialty and surgical care. The 2000 American Academy of Pediatrics (AAP) preventive pediatric health care guidelines recommend that all infants receive 10 WBVs between age 1 month and 3 years [2].

A number of studies have examined health and educational outcomes associated with WBVs during infancy and early childhood. Infants receiving the recommended number of WBVs in the first two years of life are subsequently more likely to have physician visits (to help address acute health problems) and less likely to have a visit to the emergency department [3,4]. Another study found a significant association between WBVs and kindergarten readiness [5], highlighting the social and educational benefits associated with WBVs.

Medicaid is a publicly-financed health insurance program in the U.S. for low-income infants, children, and families. State Medicaid programs are financed by federal and state dollars, but the programs are administered at the state-level. To ensure that individual Medicaid programs provide beneficiaries with a minimum set of health insurance benefits, the U.S. Early and Periodic Screening, Diagnosis, and Treatment (EPSDT) Program requires state Medicaid programs to provide WBVs to all infants according to the AAP guidelines. One study reported that 11% of Medicaid-enrolled infants in South Carolina received the recommended number of WBVs in first year of life [3]. Another study focusing on Medicaid enrollees in Philadelphia, Pennsylvania found that WBV adherence was 88% at 6 months, 47% at 12 months, 44% at 18 months, and 67% at 24 months [6]. While one study based on data from Puerto Rico reported no difference in WBV adherence between Medicaid-enrolled and privately-insured infants [7], most studies suggest that Medicaid enrollees are significantly less likely to have WBVs [8-10]. For example, significantly lower proportions of Medicaid-enrolled infants received adequate early preventive medical care compared to infants with private insurance (28% for White Medicaid-enrolled infants and 47% for White privately-insured infants, P < .01; 27% for Black Medicaid-enrolled infants and 33% for Black privately-insured infants, P < .01) [8]. Collectively, these latter studies suggest disparities in the receipt of WBVs for Medicaid-enrolled infants, which place already vulnerable infants at risk for poor health outcomes, acute health-related emergencies, and poor school performance.

Clinicians, public health officials, and policymakers recognize the importance of WBVs, but few studies have identified factors related to WBVs, especially for Medicaid-enrolled infants. The existing literature on WBVs [3,6-10] is limited for two main reasons: 1) a focus on infant and maternal factors, which overlooks health system factors such as provider reimbursement mechanism and receipt of case management services that uniquely affect Medicaid enrollees; and 2) the absence of explicit conceptual frameworks to help guide variable selection and development of WBV utilization models. We adopt an ecological conceptual model to address these key limitations and test the hypothesis that various infant, maternal, and health system factors are related to the WBVs for Medicaid-enrolled infants. In our model, WBVs are proposed as a potential infant-level mechanism by which Medicaid-enrolled infants achieve positive health and educational outcomes later in life. The knowledge generated from this study is an important step in helping policymakers and researchers develop interventions aimed at optimizing access to WBVs and improving health and educational outcomes for socioeconomically vulnerable infants enrolled in Medicaid.

## Methods

### Study design and participants

This was a secondary data analysis of enrollee-level Iowa Medicaid enrollment and medical claims data linked to birth certificate data (N = 6,322). Our analyses focused on infants: 1) born in calendar year 2000 who were enrolled in Iowa Medicaid for at least 41 continuous months from birth (to allow for an assessment of whether the infant received each of the 10 AAP-recommended WBVs); and 2) for whom we could link Medicaid and birth certificate data. We were able to match claims and birth certificate data for 96.3% of infants (n = 6,085). The University of Iowa Institutional Review Board approved this study.

### Outcome measure

The outcome measure was the total number of well baby visits (WBVs) the infant had between birth to age 41 months (range: 0 to 10). WBVs were identified from each infant’s claims files using the following International Classification of Disease-Version 9-Clinical Modification (ICD-9-CM) and Current Procedural Terminology (CPT) Codes: V20.2, V70.0, V70.3, V70.5, V70.6, V70.8, V70.9, 99381, 99382, 99391, 99392, and 99432 [11]. The selected WBV ICD-9-CM and CPT Codes were based on Health Plan Employer Data and Information Set (HEDIS) specifications, which are standardized outcome measures used to assess the quality of health insurance plans.

The 2000 American Academy of Pediatrics (AAP) WBV schedule was used to assess whether an infant had each of the following 10 recommended WBVs (no/yes): 1-month, 2-month, 4-month, 6-month, 9-month, 12-month, 15-month, 18-month, 24-month, and 36-month [2]. We assessed whether the infant had a particular WBV by subtracting the infant’s date of birth from the date on which the infant had the WBV and applied previously published age ranges [9] around each WBV (Table 1). For example, an infant who had a WBV between age 7 days and less than age 1 month was classified as having had the 1-month WBV. Finally, we summed the total number of WBVs that each infant had during the 41-month study period.

**Table 1:** American Academy of Pediatrics (AAP) well baby visit periodicity schedule from 2000 and operationalization of study outcome measure (total number of well baby visits received by age three years)

**2000 AAP well baby visit periodicity schedule**	Age ranges*
1 month	7 days to <1 month
2 month	1 month to <3 months
4 month	3 months to <5 months
6 month	5 months to <8 months
9 month	8 months to <11months
12 month	11 months to <14months
15 month	14 months to <17 months
18 month	17 months to <20 months
24 month	20 months to <30 months
36 month	30 months to 41 months

*Byrd et al. 1999 [9].

### Conceptual model and predictor variables

We used an ecological model of health [12] and existing literature [7,10,13-16] to construct a preliminary explanatory model of factors related to the receipt of WBVs for Medicaid-enrolled infants. While not tested in the current study, WBVs represent an important infant-level variable associated with positive outcomes later in life such as increased use of office-based preventive medical care services, decreased use of the emergency department management of acute health problems, and improved school readiness [3-5]. We hypothesize that the following 12 infant, maternal, and health system variables are related to the receipt of WBVs for Medicaid-enrolled infants.

#### Infant variables

There were two infant variables: having a chronic health condition during the first 16 months of life (no/yes) [14] and eligibility for Medicaid through the Supplemental Security Income (SSI) Program (no/yes). We identified chronic health conditions using ICD-9-CM, CPT, and Healthcare Common Procedure Coding System (HCPCS) codes that have been published previously [17]. Examples of chronic health conditions include premature birth, low birth weight, ventilator use, gastrostomy, tracheotomy, infantile seizures, and newborn apnea. SSI eligibility is based on the presence of a disability documented by a physician or health provider. In our model, SSI is a proxy for chronic health condition severity and was measured by assessing whether the infant gained eligibility for Medicaid through the SSI Program for at least 6 months in the first year of life.

#### Maternal variables

There were seven maternal variables: maternal age at time of child’s birth (<18; 18 to 20; 21 to 29; ≥30 years) [16]; race (White; non-White) [13]; education (less than high school; high school or more) [10]; and marital status (married; other) [13]. We included the number of prenatal visits (quartiles) based on the premise that mothers who use greater amounts of prenatal care are more likely to ensure that their child receives WBVs [15]. We also included two exploratory self-reported maternal factors: alcohol use and maternal smoking status during pregnancy (no/yes).

#### Health system variables

There were three health system variables. The first was county-level rurality, a four-level variable based on the U.S. Department of Agriculture Rural and Urban Continuum Codes (rural; urban non-adjacent to metropolitan; urban adjacent to metropolitan; metropolitan). Rurality is an important health system factor in terms of the local supply and characteristics of health care providers [18,19]. Previous studies have examined rurality as a potential factor related to WBVs [13] and visits to pediatricians [19]. The remaining two health system factors were exploratory and relevant specifically to Medicaid enrollees: enrollment in a health maintenance organization (HMO) plan (no/yes) and recipient of MediPASS primary care case management services (no/yes). Medicaid HMOs in Iowa started in 1986 as a pilot project in one county and expanded to 43 of 99 counties [20]. HMOs provide a capitation fee (a pre-established monthly reimbursement based on patient encounter) rather than fee-for-service (FFS), in which reimbursement is directly related to intensity of services provided. MediPASS was implemented in 1990 in seven counties and has since expanded to 93 Iowa counties [20]. It is a gate-keeper model in which enrollees are assigned a primary care case manager. Coverage of health care services in HMOs and MediPASS in Iowa is the same as in traditional FFS Medicaid.

### Statistical analyses

We generated descriptive statistics on the study population. Next, we ran standard regression diagnostics on the outcome measure (total number of WBVs) to evaluate the normality assumption and heteroscedasticity (both of which were acceptable) and the presence of outliers using delta beta plots (no outliers present). We also evaluated bivariate relationships between the 12 potential predictor variables and the outcome measure (total number of WBVs) with one-way ANOVA (α = 0.05). Finally, we ran multiple variable linear regression models to generate a final explanatory model on the factors related to WBVs for Medicaid-enrolled infants. As part of exploratory analyses, we included an interaction term between the HMO and MediPASS variables to examine the role of being in a capitated plan with access to case management services. We limited our interaction analyses to the two health system covariates with the greatest potential policy relevance (HMO and primary care case management) because of the effect these factors have in containing pediatric health care costs [21,22]. To address potential model misspecification, we ran our models with and without robust standard errors. Because the two approaches yielded similar results, we report findings from model with robust standard errors. All analyses were completed using PASW Statistics version 18.0 for Windows (Chicago, IL).

## Results

### Descriptive statistics

One-in-three infants had a chronic health condition and 2.1% were eligible for Medicaid through the Supplemental Security Income (SSI) Program (Table 2). Over 35% of infants had a mother younger than age 21 years and 75.4% of mothers completed less than high school. One-in-three mothers were married. The mean number of prenatal visits was 11.5 (standard deviation: 3.9) (data not shown). Nearly 3% of mothers reported using alcohol and 35.1% reported smoking during pregnancy. Most infants lived in a metropolitan county (55.5%). Nearly 40% were in a HMO plan and 31.7% received Medipass primary care case management services. All infants had at least one WBV and 6.6% received all 10 WBVs (data not shown). The mean number of WBVs was 6.4 (SD: 2.2). WBV utilization rates ranged from 42.0% (9-month WBV) to 87.0% (2-month WBV).

**Table 2:** Descriptive statistics and one-way anova results between predictor variables and well baby visits received by age three years for Medicaid-enrolled infants (n = 6,085)

**Variable**	**n**	**%**	**Mean number of well baby visits**	**P-value**
Infant variables				
Had a chronic health condition in the first 16 months of life				P < .0001
No	4056	66.7	5.98	
Yes	2029	33.3	6.61	
Eligible for Medicaid through the Supplemental Security Income (SSI) Program				P = .038
No	5958	97.9	6.20	
Yes	127	2.1	5.80	
Maternal variables				
Age (years)				P < .0001
<18	510	8.4	6.52	
18 to 20	1641	27.0	6.26	
21 to 29	3056	50.2	6.15	
≥30	877	14.4	6.00	
Race				P < .0001
White	5235	87.5	6.23	
Non-White	760	12.5	5.91	
Education				P = .006
Less than high school	4586	75.4	6.24	
High school or more	1443	23.7	6.06	
Missing	56	0.9	-	
Marital status				P = .010
Married	1766	29.0	6.08	
Other	4319	71.0	6.24	
Prenatal visit quartiles (number of prenatal visits)				P = .002
1st quartile (less than 10 visits)	1435	23.6	6.00	
2nd quartile (10 to 11 visits)	1335	21.9	6.19	
3rd quartile (12 to 13 visits)	1561	25.7	6.24	
4th quartile (14 or more visits)	1535	25.2	6.27	
Missing	219	3.6	-	
Self-reported maternal alcohol use during pregnancy				P = .797
No	5801	95.3	6.19	
Yes	158	2.6	6.15	
Missing	126	2.1	-	
Self-reported maternal smoking status during pregnancy				P = .065
No	3848	63.2	6.23	
Yes	2137	35.1	6.12	
Missing	100	1.6	-	
Health system variables				
Rurality				P = .895
Rural	355	5.8	6.14	
Urban non-adjacent to metropolitan	1172	19.3	6.18	
Urban adjacent to metropolitan	1183	19.4	6.23	
Metropolitan	3375	55.5	6.19	
Enrollment in a health maintenance organization (HMO) plan				P < .0001
No	3673	60.4	6.75	
Yes	2412	39.6	5.35	
Recipient of MediPASS primary care case management services				P < .0001
No	4155	68.3	5.84	
Yes	1930	31.7	6.96	

### Bivariate statistics

Infants with a chronic health condition had a greater number of WBVs than those without a chronic health condition (6.61 and 5.98, respectively, P < .0001) (Table 2). There was a negative relationship between maternal age and mean number of WBVs (P < .0001) and a positive relationship between maternal prenatal visits and WBVs (P = .002). Infants in a health maintenance organization (HMO) plan had significantly fewer WBVs than those not in a HMO plan (P < .0001) whereas recipients of MediPASS primary care case management services had significantly more WBVs than non-MediPASS infants (P < .0001).

### Linear regression model

The following factors were positively associated with the total number of WBVs (Table 3): infants with a chronic health condition (ß = 0.51, P < .0001), not being married (ß = 0.20, P = .002), greater number of maternal prenatal visits (reference group, less than 10 visits; 10 to 11 visits, ß = 0.24, P < .001; 12 to 13 visits, ß = 0.23, P = .002; 14 or more visits, ß = 0.28, P = .001), and receipt of MediPASS services (ß = 0.48, P < .0001). Eligibility through the SSI Program (ß = −0.70, P = .001), older maternal age (ß = −0.35, P = .003 to ß = −0.27, P = .004), maternal education of high school or more (ß = −0.18, P = .005), maternal smoking (ß = −0.13, P = .018), and enrollment in a HMO plan (ß = −1.15, P < .0001) were negatively associated with WBVs. There was a significant interaction between HMO enrollment and receipt of MediPASS services (ß = 0.66, P = .015). Maternal race, maternal alcohol use, and rurality were not related to WBVs.

**Table 3:** Final linear regression model on factors related to the number of of well baby visits received by age three years for Medicaid-enrolled infants

**Variable**	**Beta coefficient**	**Standard error**	**95% CI**	**P-value**
Infant variables				
Had a chronic health condition in the first 16 months of life				P < .0001
No	ref	-	-	
Yes	0.51	0.06	0.40, 0.62	
Eligible for Medicaid through the Supplemental Security Income (SSI) Program				P = .001
No	ref	-	-	
Yes	−0.70	0.22	−1.13, −0.28	
Maternal variables				
Age (years)				
<18	ref	-	-	-
18 to 20	−0.17	0.10	−0.35, 0.22	P = .084
21 to 29	−0.27	0.09	−0.45, −0.09	P = .004
≥30	−0.35	0.12	−0.58, −0.12	P = .003
Race				P = .792
White	ref	-	-	
Non-White	−0.02	0.08	−0.18, 0.13	
Education				P = .005
Less than high school	ref	-	-	
High school or more	−0.18	0.07	−0.31, −0.06	
Marital status				P = .002
Married	ref	-	-	
Other	0.20	0.06	0.08, 0.33	
Prenatal visit quartiles (number of prenatal visits)				
1st quartile (less than 10 visits)	ref	-	-	-
2nd quartile (10 to 11 visits)	0.24	0.08	0.09, 0.39	P < .001
3rd quartile (12 to 13 visits)	0.23	0.08	0.06, 0.38	P = .002
4th quartile (14 or more visits)	0.28	0.08	0.13, 0.43	P = .001
Self-reported maternal alcohol use during pregnancy				P = .081
No	ref	-	-	
Yes	0.26	0.15	−0.03, 0.54	
Self-reported maternal smoking status during pregnancy				P = .018
No	ref	-	-	
Yes	−0.13	0.06	−0.25, −0.02	
Health system variables				
Rurality				
Rural	ref	-	-	-
Urban non-adjacent to metropolitan	0.04	0.07	−0.10, 0.17	P = .575
Urban adjacent to metropolitan	0.05	0.07	−0.09, 0.18	P = .506
Metropolitan	−0.03	0.12	−0.26, 0.21	P = .834
Enrollment in a health maintenance organization (HMO) plan				P < .0001
No	ref	-	-	
Yes	−1.15	0.07	−1.28, −1.01	
Recipient of MediPASS primary care case management services				P < .0001
No	ref	-	-	
Yes	0.48	0.07	0.34, 0.63	
Multiplicative interaction term between HMO and MediPASS variables	0.66	0.27	0.13, 1.18	P = .015

## Discussion

This is the first study to adopt an ecological model to examine the factors related to the receipt of well baby visits (WBVs) for Medicaid-enrolled infants. We tested the hypothesis that various infant, maternal, and health system factors would be associated with the number of recommended WBVs received during the first 41 months of life for Medicaid-enrolled infants. The current study supports this hypothesis. We have three main findings.

The first finding is that both infant variables (having a chronic health condition and Medicaid eligibility through the Supplemental Security Income [SSI] Program) were significantly associated with WBVs. One-in-three Medicaid-enrolled infants in our study had a chronic health condition, which is higher than the national prevalence of children with special health care needs (17.6% to 19.3%) [23] but in line with higher chronic health condition prevalence rates for Medicaid-enrolled children [24]. The results on chronic conditions are consistent with a related study reporting that children with special health care needs under age five years were significantly more likely to have a well child visit in the previous year than those without a special health care need [25]. There are three possible explanations for this finding. First, infants with chronic health conditions may have greater need for ambulatory care because of their medical conditions. These infants may require more follow-up care after acute illnesses and are likely to require prescription medications. Second, caregivers of infants with chronic health conditions may be more vigilant about compliance with the recommended WBV schedule. Third, medical providers may emphasize the need for medically-vulnerable infants to be seen regularly for preventive care to ensure proper monitoring and treatment compared to healthy infants. Our proxy measure of chronic condition severity (eligibility for Medicaid through SSI) was associated with fewer WBVs, which suggests that infants with the most severe chronic conditions are less likely to receive WBVs. Children in SSI have intellectual or developmental disabilities or acquired cognitive deficits, both of which can be barriers to health care services. What is unknown is whether fewer WBVs translate to greater levels of unmet health care needs. Future work should examine the relationship between WBVs and unmet need among Medicaid-enrolled infants and clarify how caregivers define unmet need (e.g., primary versus specialty care). This evidence could then be used to develop targeted interventions to ensure that all Medicaid-enrolled infants receive appropriate levels of preventive medical care.

The second finding is that a number of maternal factors were associated with WBVs for Medicaid-enrolled infants. Maternal age and education were negatively associated with WBVs. These findings are inconsistent with those from a study of a cohort of Medicaid-eligible infants in Philadelphia in which there was no significant relationship between WBVs and maternal age or education [6]. One possible explanation of the inconsistency regarding maternal age is underlying differences in sociodemographic characteristics. While the mean maternal age in our study and this other study was similar (23 years), there were three important differences. The latter study focused on a small population of urban Medicaid-enrolled infants in Philadelphia whose mothers were mostly African American (92%), high school graduates (73%), and unmarried (88%). Our study included mainly White mothers (87.5%) in Iowa, most of whom did not complete high school (75%) with a higher proportion of whom were married (29%). In both studies, prenatal visits were significantly associated with WBVs, suggesting that mothers who use preventive medical care are more likely to ensure that their children also utilize recommended preventive care. In regards to the negative relationship between education and the number of WBVs, it may be that higher levels of education are associated with compensatory behaviors that result in fewer WBVs. For example, mothers who are familiar with the specific types of preventive care provided during WBVs may chose to skip particular WBVs (e.g., WBVs in which immunizations are not provided) because their infant is otherwise perceived to be healthy. Additional research is needed on the effects of maternal education on preventive health utilization patterns for Medicaid-enrolled infants. Furthermore, maternal alcohol use during pregnancy was not significantly associated with WBVs. This is consistent with null findings from a population of privately-insured infants in Kaiser Permanente Northwest [26] and infants in Canada [27] but inconsistent with findings reported by Minkovitz and colleagues [28]. This latter study focused on the post-partum period, whereas the studies with null findings examined the pre-partum period, which may explain these inconsistencies. Smoking during pregnancy was negatively associated with WBVs, suggesting that mothers who smoke during pregnancy may be less likely to seek WBVs for their children after birth. Researchers should continue to elucidate the relationship between maternal factors and infant health utilization.

The third finding is that health system variables were important correlates of WBVs for Medicaid-enrolled infants. In terms of main effects, enrollment in a HMO plan was negatively associated with WBVs whereas being a recipient of MediPASS services was positively associated with WBVs. These findings are new contributions to the pediatric health services literature. However, we also detected an interaction between these two health system factors. Enrollment in a HMO and receipt of case management services was negatively associated with WBVs ([ß_HMO_ = −1.15] + [ß_MediPASS_ = 0.48] + [ß_HMO × MediPASS_ = 0.66] = −0.01). Previous work suggests that Medicaid SSI enrollees in managed care plans have significantly fewer unmet health care needs than those in FFS plans [29]. As States continue to implement managed care within Medicaid programs, case management services may be a way to ensure that enrollees’ health needs are met and to reduce costs (by reducing demand for WBVs). Rurality was not associated with WBVs, which is consistent with bivariate analyses conducted by Freed and colleagues [13]. Future work should continue to assess the relationship between health system factors and individual use of health care services with an emphasis on appropriate use of services based on need.

**Figure 1:**
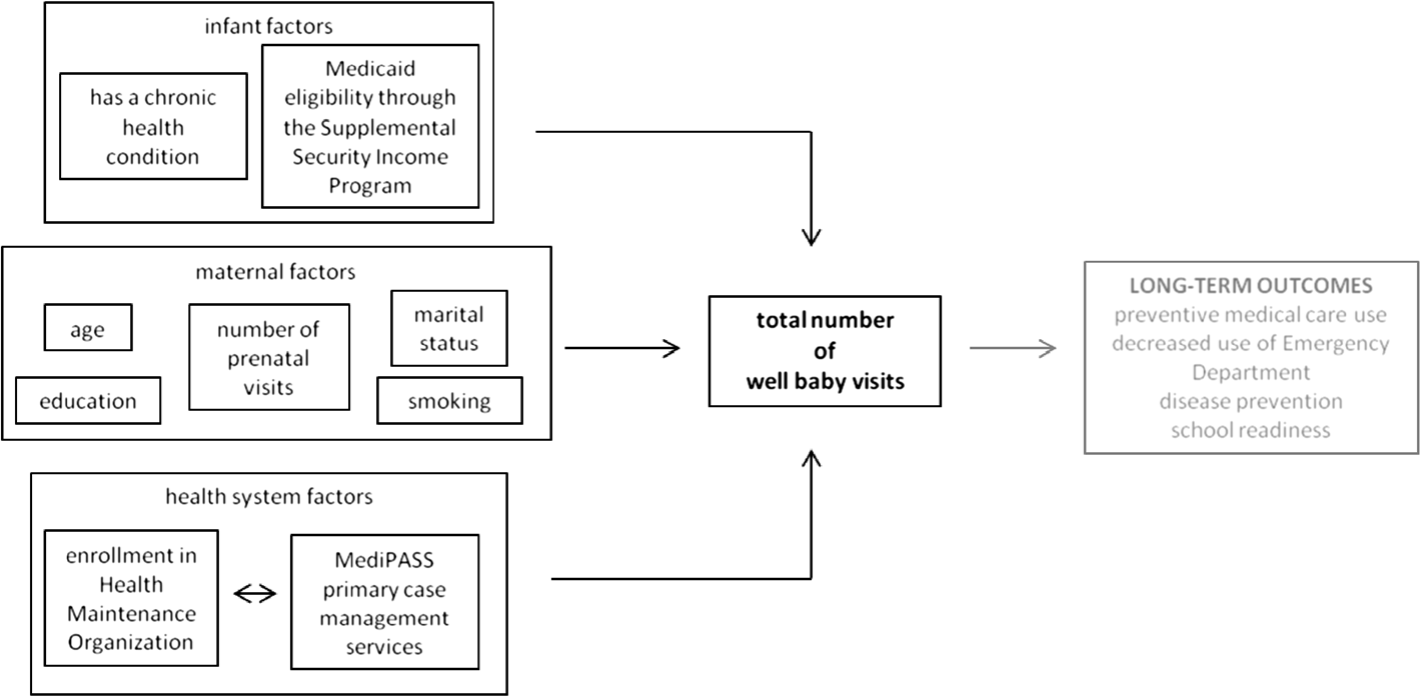
Preliminary explanatory model on factors related to well baby visits (WBS) by age three years for Medicaid-enrolled infants.

Broadly, our findings support a preliminary explanatory model on factors related to the well baby visits for Medicaid-enrolled infants (Figure 1). This model includes infant, maternal, and health system factors associated with WBVs. Some of these factors are immutable (e.g., having a chronic health condition) and could form the basis for targeted interventions aimed at high-risk subgroups whereas others are modifiable (e.g., primary care case management services) and could be implemented at the system-level to benefit all Medicaid-enrolled infants. WBVs, in turn, are hypothesized correlates of positive health and educational outcomes, though these associations were not evaluated in the current study. Missing from our preliminary model are the social determinants of health (e.g., disadvantage, deprivation, segregation, financial hardship, social capital) that could be measured at the community- or neighborhood-level as well as other area-level factors that could influence receipt of WBVs such as poverty, availability of and proximity to medical providers, and transportation-related resources. Future research should continue to test and refine explanatory models that identify the determinants and outcomes associated with WBVs, which will help guide efforts to ensure that all Medicaid-enrolled infants receive optimal preventive medical care in early childhood.

Our study had five main limitations. The first is limited accuracy of birth certificate data especially regarding the number of prenatal visits reported by mothers [30]. To address this limitation, we adopted prenatal visit quartiles in our models rather than number of prenatal visits. It is also possible that mothers underreported on measures such as smoking and alcohol use because of social desirability bias, which could have influenced the accuracy of our measurement of these two variables. Future research should incorporate methods that minimize and account for social desirability bias. In addition, validated primary data collection methods could be used to measure maternal depression, maternal stress, and social support. The second limitation is that there were differences between infants in the study and infants for whom we were unable to match Medicaid and birth certificate data, which may limit external generalizability of study findings. For instance, the 3.7% of unmatched infants (excluded from the analyses) were less likely to have a chronic health condition and more likely to be non-White. We did not impute missing birth certificate data because validated methods for imputing missing data are unavailable and simple imputation techniques are likely to result in biased estimates [31]. Future work should develop birth certificate data imputation methods like those developed for imputing gestational age [32]. The third limitation is the lack of data on unmet primary health care needs. We assumed that infants with a greater number of WBVs would have fewer unmet needs, but this hypothesis requires formal evaluation. Future studies should incorporate methods to collect primary data from medical providers and caregivers to assess the extent to which WBVs are related to unmet primary care needs. The fourth limitation is that we assumed accurate coding by physicians to reflect WBVs as prevention-oriented visits though it is possible that acute care needs are also addressed during WBVs. The fifth limitation is that our study focused on a cohort of Medicaid enrollees born in calendar year 2000. There is no reason to believe that the factors related to WBVs have changed significantly during the past 10 years. However, additional studies on more recent cohorts of Medicaid-enrolled infants would provide insight on the extent to which the factors related to WBVs have changed over time.

## Conclusions

Our findings suggest that multiple infant, maternal, and health system variables are significantly related to the number of WBVs in the first three years of life received by Medicaid-enrolled infants in our study. Improving prenatal care use for women in Medicaid and expanding enrollment in case management programs for newborn Medicaid enrollees could help to increase the number of WBVs received by Medicaid-enrolled infants. There are also opportunities to develop and test WBV interventions targeted at Medicaid-enrolled infants in the Supplemental Security Income Program. Future research should continue to evaluate the determinants of and outcomes associated with WBVs, the relationship between receipt of WBVs and unmet health care needs, and explanatory models that can guide interventions and policies aimed at ensuring that all Medicaid-enrolled infants receive optimal preventive medical care in early childhood.

## Competing interests

The authors declare that they have no competing interests.

## Authors’ contributions

DLC conceptualized and designed the study, analyzed the data, and led the writing. ETM obtained and assimilated the datasets. All authors participated in the design of the study, helped to interpret findings, helped draft the manuscript. All authors read and approved the final manuscript.

## Pre-publication history

The pre-publication history for this paper can be accessed here:

http://www.biomedcentral.com/1471-2431/13/158/prepub
